# Design and Development of a Bio-Inspired UHF Sensor for Partial Discharge Detection in Power Transformers

**DOI:** 10.3390/s19030653

**Published:** 2019-02-05

**Authors:** Luiz A. M. M. Nobrega, George V. R. Xavier, Marcus V. D. Aquino, Alexandre J. R. Serres, Camila C. R. Albuquerque, Edson G. Costa

**Affiliations:** Department of Electrical Engineering, Universidade Federal de Campina Grande, Aprigio Veloso 882, Campina Grande 58429-900, Brazil; george.xavier@ee.ufcg.edu.br (G.V.R.X.); marcus.aquino@ee.ufcg.edu.br (M.V.D.A.); alexandreserres@dee.ufcg.edu.br (A.J.R.S.); camila.albuquerque@ee.ufcg.edu.br (C.C.R.A.); edson@dee.ufcg.edu.br (E.G.C.)

**Keywords:** bio-inspired, partial discharge, antenna, UHF sensor, power transformer, monitoring

## Abstract

In this paper, the design and development of a bio-inspired UHF sensor for partial discharge detection in power transformers is presented. The UHF sensor was developed for external use in dielectric windows of power transformers. For this purpose, a microstrip antenna was designed with a radiating element shape based on the leaf of the *Jatropha mollissima (Pohl) Baill* plant. Then, an epoxy coating and an aluminium enclosure were developed to protect the antenna against corrosion and to provide mechanical support, external noise immunity, and a lifetime compatibility with power transformers. In order to verify the electrical parameters of the developed sensor, measurements of the gain and the reflection coefficient were performed in an anechoic chamber. Lastly, the antenna sensitivity for denominated partial discharge (PD) detection was compared with the IEC 60270 standard method. For this purpose, simultaneous tests were carried out in a partial discharge generator setup, composed of an oil cell with needle-plane electrodes. The experimental tests demonstrated the effectiveness of the sensor for detecting PD signals with apparent charge values higher than 35 pC.

## 1. Introduction

Electrical insulation systems are one of the main components of high-voltage equipment. In general, these insulation systems have higher dielectric strength than air, allowing them to support high-voltage levels with a shorter distance between electrodes. In this way, the system becomes more compact, reducing space and costs. However, the insulating material is subjected to high electrical fields, which excites the electrical charges inside the insulation. The acceleration of these charges by the electrical field may cause electron avalanche, in which parts of the insulation become conductive. When this occurs, small internal discharges appear, known as denominated partial discharges (PD). These discharges, although initially of a small magnitude, are by nature an evolutionary process that cause chemical decomposition and material erosion. As a consequence, the damaged area can increase in size and cause the insulation to breakdown. Therefore, damage to the insulation of electrical equipment must be recognized in its initial stages, PD detection being the most common method used for this purpose.

As far as PD detection and measurement mechanisms are concerned, several techniques have been used for monitoring electrical equipment, such as the method defined by the IEC 60279 standard [1] and acoustic [2,3], optical [4,5], and chemical [6] detection methods. In addition, one technique that has received attention in the last years uses general UHF sensors to detect, locate, and characterize PD activity [7,8,9,10,11,12,13,14].

Initially, the UHF measurement was used for the diagnosis of a gas insulated substations (GIS) [15], becoming an economically viable online monitoring method for PD measurement [16]. As a result of the consolidation of UHF monitoring in GIS, particularly because of its good immunity to external noise and the possibility of locating defects, in 1997, researchers began to apply the method in power transformers [17]. Since then, countless investigations have been developed, and the applicability of UHF PD measurement in transformers has been promising. Indeed, currently, the Cigre [18] recommends the manufacturing of power transformers with factory adaptation for UHF PD monitoring, whose first models have already been presented in the literature [19].

Among the UHF sensors that can be applied for PD detection, microstrip antennas are highlighted because of some interesting practical features, such as their low cost, ease of installation and manufacture, attractive radiation patterns, and wideband. However, the microstrip antennas with classical radiating element (patch) shapes (such as, rectangles, circles, triangles, squares, and others) designed to operate in the UHF range would assume relatively large dimensions [20,21,22,23,24], limiting their practical application for PD detection in power transformers.

According to References [25,26], the operating frequency of microstrip antennas is directly related to the perimeter of the radiating element. The greater the perimeter, the lower the operating frequency. Therefore, techniques applied in antenna miniaturization mainly seek geometries that maximize the patch perimeter. These optimized geometries can be addressed in the shapes of living beings found in nature, developed in order to provide greater efficiency regarding the ability to survive. Hence, aiming to achieve a better radiating efficiency, bio-inspired antennas use the structure of plants or animals as a basis for their design. Moreover, bio-inspired designs generally present a higher perimeter/area ratio, allowing an antenna size reduction. Furthermore, bio-inspired geometries provide a higher density of current concentrations on the transmission line, resulting in a higher gain than classical microstrip antenna shapes, essential for the detection of low intensity PD pulse signals [27].

Studies involving bio-inspired antennas have been receiving great attention in several fields, such as ultrawideband operation [25], band-stop purposes [28], coplanar waveguide antennas [29], 2G, 3G, and 4G operation [27], and wireless systems [26]. Some studies applied other miniaturizing techniques in the development of UHF sensors for PD detection, such as fractal shapes as well as [30,31,32,33] other alternative shapes [34,35,36]. However, narrow bandwidth sensors were produced, failing in the full coverage of the PD activity frequency range. In contrast, with the simultaneous application of a bio-inspired geometry design and the truncated ground plane bandwidth enhancement technique, it is possible to develop a UHF sensor that presents a compact size (necessary for the application) and with enough bandwidth for the PD detection in power transformers.

In order to contribute to the state-of-the-art techniques in transformer monitoring, this paper presents the design and development of a bio-inspired UHF sensor for PD monitoring of power transformers. The sensor is developed for adaptation in dielectric windows, and for this purpose, an aluminum enclosure was developed in order to protect the antenna against corrosion and to provide mechanical support, external noise immunity, and a lifetime compatibility with power transformers.

This paper is organized as follows: Section 2 presents an overview of PD UHF transformer monitoring. In Section 3, the detailed design of the UHF sensor proposed in this research is described. In Section 4, the experimental tests carried out for validation of the designed UHF sensor are presented, while the discussions and conclusion are presented in Section 5 and Section 6, respectively.

## 2. UHF Monitoring of Power Transformers

**Figure 1:**
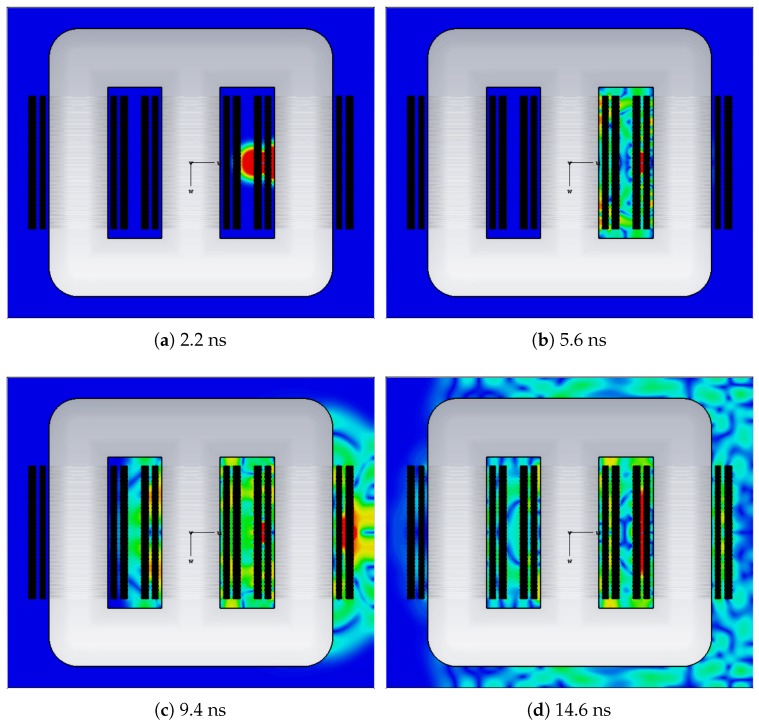
Radiation of electromagnetic waves in a power transformer.

The UHF monitoring principle is based on the electromagnetic waves radiated by the PD pulse, which has a rise time of less than a nanosecond [37]. The electromagnetic waves emitted by the PD pulse are propagated in all directions with frequencies that vary depending on the phenomenon, type of defect, and propagation material. In power transformers, the radiation typically assumes a frequency range from 0 to 1500 MHz [17,38]. To exemplify the phenomenon, Figure 1 presents a simulation of a UHF propagation in a power transformer in four different moments. As can be seen, the electromagnetic waves are radiated from the PD source until they reach the equipment tank. In this way, UHF sensors can be installed on the tank of the the equipment to detect, identify, and locate PD sources.

The UHF PD monitoring in power transformers has three main advantages. The first one is the high immunity to external noise due to the shielding effect of the transformer tank. The second one is the immunity to detection of corona discharge signals, since these signals do not have appreciable energy at UHF frequencies [39]. The third advantage of the method is its high sensitivity due to the moderately attenuated propagation in the transformer oil [40], in a way that only one sensor is required for the continuous monitoring of the equipment [13] and four sensors for locating the PD sources [10].

For the UHF monitoring of PD in transformers, several types of sensors can be used, being normally classified as internal [41], external [13,14,19], and valve sensors [7,42]. Hereafter, we describe the installation of external sensors for use in dielectric windows, the object of study of this paper.

**Figure 2:**
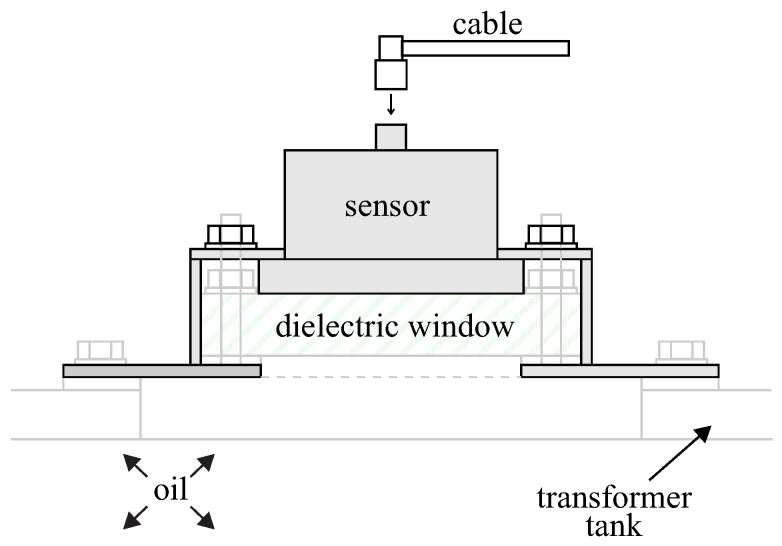
UHF sensor and window assembly on a transformer hatch cover [19].

The external window sensors are installed in apertures in the equipment tank, against dielectric windows retrofitted or constructed for this purpose. Figure 2 presents a cross-section through an inspection hatch with a UHF sensor installed in a dielectric window. As can be observed, it is necessary to insert a dielectric material between the internal part of the transformer and the sensor to allow the electromagnetic waves to find a path to the external environment (where the sensor is located). According to Reference [19], the choice of the dielectric material is not especially critical from the perspective of electromagnetic properties. Mechanical, chemical, and lifetime properties must be dominant choice factors. With a suitable mechanic design, the window is not exposed to the external environment and must keep the oil pressure in a normal operational range, being always covered by the sensor or by a protection plate. For the dielectric materials, either polytetrafluoroethylene (PTFE)—which is chemically inert and impermeable to moisture—or several types of epoxy resins can be used [19].

## 3. UHF Sensor Design

The UHF sensor designed in this research is composed of three main parts: (1) one microstrip antenna with bio-inspired technology, to capture the UHF signal radiated from PD sources; (2) an epoxy coating, which provides the sensor with protection against corrosion, mechanical support, and high temperature tolerance; and (3) an electromagnetic shield, in order to guarantee immunity to external noise. Next, we present the procedures performed to obtain the final version of the developed sensor.

### 3.1. Bio-Inspired Microstrip Antenna

The first step in the development of the sensor is the design and manufacture of the bio-inspired microstrip antenna. For that, the initial design parameters were a central resonance frequency of 900 MHz and a bandwidth of 1200 MHz. A FR4 dielectric substrate ($\varepsilon_{r}$ = 4.4) was used with a thickness of 1.6 mm. The shape of the patch antenna was based on the leaf of the *Jatropha mollissima (Pohl) Baill* plant and the ground plane was truncated along the length of the transmission line to increase the antenna bandwidth [43]. Among the several plant shapes analyzed, the *Jatropha mollissima (Pohl) Baill* provided a good perimeter/area ratio, since the antenna patch fills a great area within the compact dimensions required for the installation in the dielectric window, and conferred enough bandwidth and gain results for PD detection applications in power transformers as is presented in the coming sections.

In the design of the patch antenna, the perimeter (*p*) of the bio-inspired geometric shape was calculated in agreement with the lowest resonance frequency ($f_{L}$) of PD occurrence (300 MHz) according to Equations (1) and (2) [25]:

(1) $$\varepsilon_{ref}=\frac{\varepsilon_{r}+1}{2}+\frac{\varepsilon_{r}-1}{2}{\left[1+12\frac{h}{W}\right]}^{-1/2},\;\mathrm{for}\;W/h>1,$$

(2) $$f_{L}\left(GHz\right)=300/\left(p\sqrt{\varepsilon_{ref}}\right),$$

where *h* represents the dielectric substrate thickness and *W* the microstrip width.

**Figure 3:**
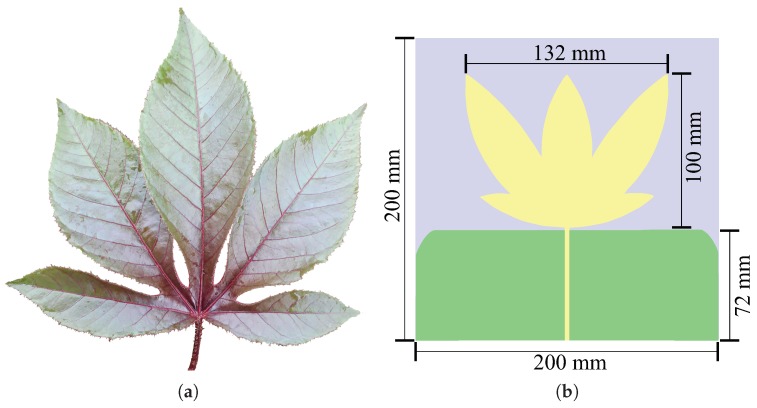
(**a**) Graphical representation of the *Jatropha mollissima (Pohl) Baill* leaf and (**b**) the designed bio-inspired antenna.

In order to obtain an optimized structure in terms of gain, size, and coefficient of reflection, geometric parameter sweeps, such as, width and length of the leaves, as well the distance and opening angle between them were calculated. The final detailed dimensions of the described bio-inspired antenna and the leaf of the *Jatropha mollissima (Pohl) Baill* plant are summarized in Figure 3.

**Figure 4:**
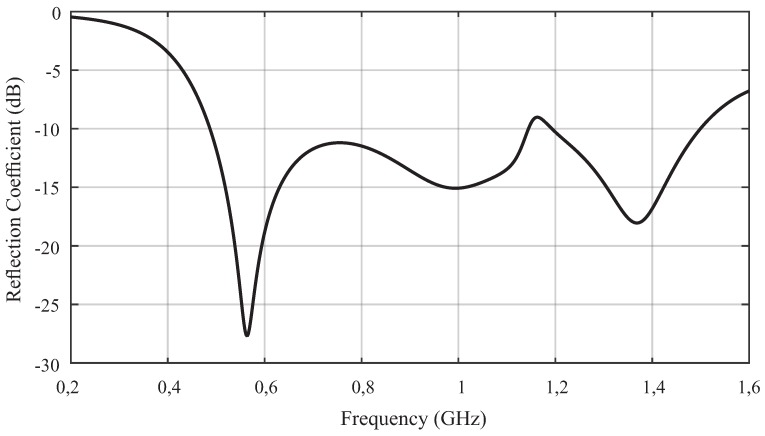
Simulated reflection coefficient for the bio-inspired antenna.

To obtain the main parameters of the antenna, simulations were performed in the high frequency structure simulator (HFSS) from ANSYS. The first simulation result analyzed was the antenna reflection coefficient, as shown in Figure 4.

As can be seen in Figure 4, the bio-inspired antenna presented an operating band from 487 to 1145 MHz and 1194 to 1497 MHz, with a reflection coefficient less than −10 dB. Therefore, the bio-inspired antenna was able to provide a satisfactory bandwidth over most of the range of interest for PD detection (300 to 1500 MHz).

**Figure 5:**
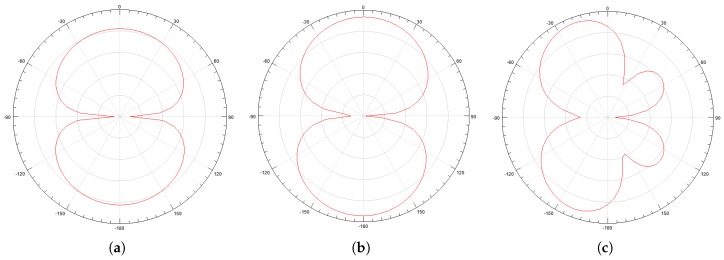
Antenna radiation pattern for: (**a**) 487 MHz; (**b**) 992 MHz; (**c**) 1497 MHz.

**Figure 6:**
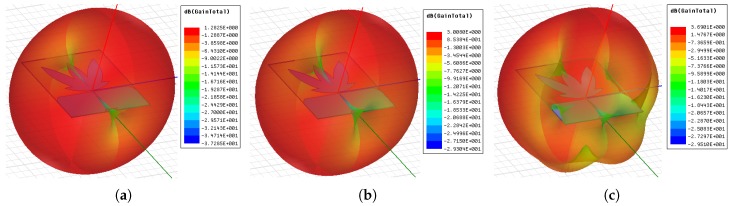
3D directive gain pattern for: (**a**) 487 MHz; (**b**) 992 MHz; (**c**) 1497 MHz.

In addition to the reflection coefficient, the antenna radiation pattern and gain for the lower (487 MHz), central (992 MHz), and upper (1497 MHz) frequencies were obtained, as shown in Figure 5 and Figure 6. From the radiation patterns, it is possible to verify that most of the energy is radiated in the direction of interest for PD detection, corresponding to 0° in the radiation pattern. The mean gain obtained in the direction of maximum radiation was 2.69 dBi.

### 3.2. Electromagnetic Shield

**Figure 7:**
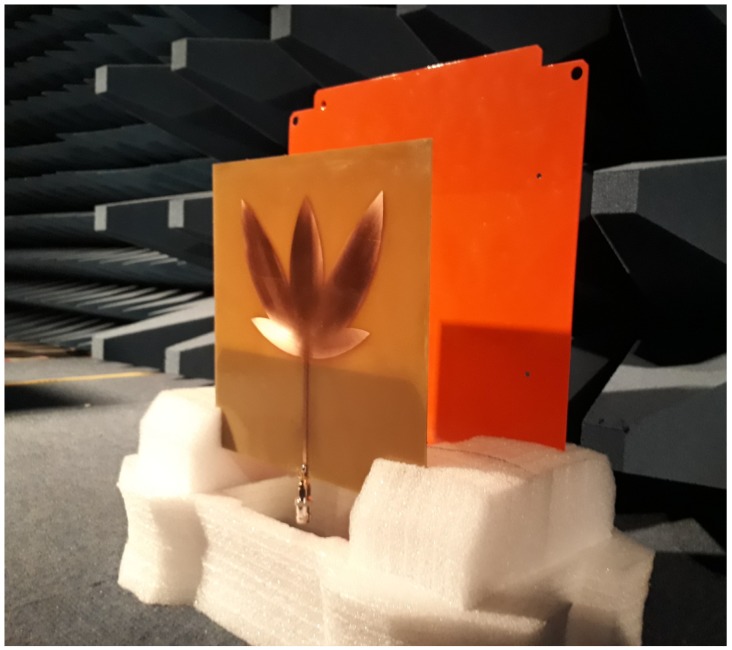
Experimental setup for the electromagnetic shielding test.

**Figure 8:**
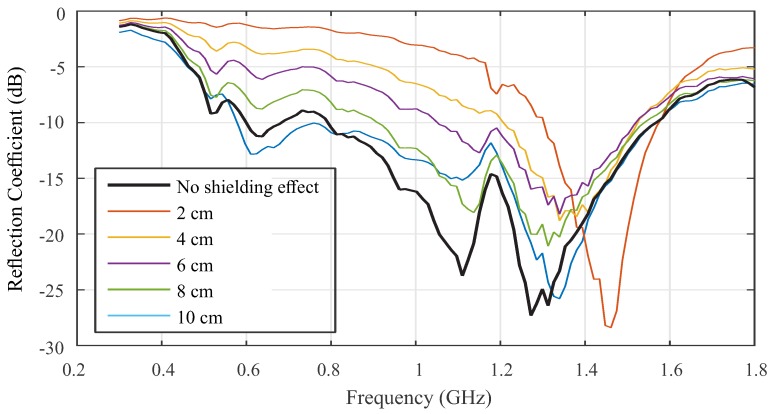
Electromagnetic shielding effect over the bio-inspired reflection coefficient.

Evaluations of the electromagnetic shielding effect on the antenna reflection coefficient were performed in laboratory. For that, the antenna was manufactured in a LPKF ProtoMat S103 circuit board plotter. Then, a metal plate was moved towards the antenna ground plane at fixed distances of 2, 4, 8, and 10 cm and the reflection coefficient was measured for each distance. A photograph of the experimental setup is shown in Figure 7, which used a vector network analyzer (VNA) E5071C (9 MHz–8.5 GHz) from Keysight Technologies for measurement. The antenna reflection coefficient for each measurement is shown in Figure 8.

From the results, it is observed that the closer the metal structure is to the antenna, the more degenerate the measured reflection coefficient. The best results were obtained with the metal plate at a distance of 8 and 10 cm. In the first scenario, it is possible to verify a reflection coefficient below −5 dB in the frequency range of interest. In the second scenario, the effect of the metal plate was negligible, so that the reflection coefficient presented values below −10 dB in the range of 600 MHz to 1.5 GHz.

### 3.3. Proposed UHF Sensor

**Figure 9:**
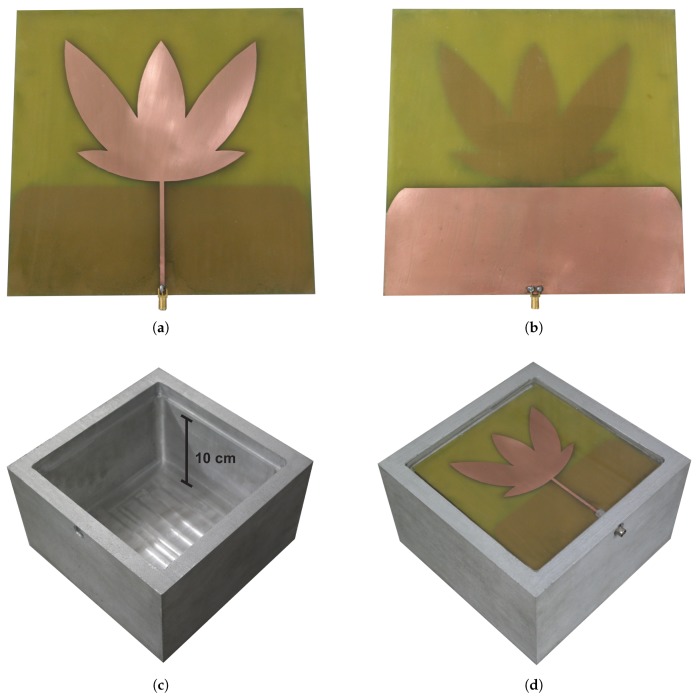
Components and assembly of the UHF sensor. (**a**) Radiating element of the bio-inspired antenna. (**b**) Ground plane on the other side of the antenna. (**c**) Aluminum sensor enclosure. (**d**) Complete assembly of the UHF sensor.

After the measurements, the microstrip antenna was encapsulated in epoxy resins with a thickness of 20 mm to provide protection against corrosion, mechanical support, and a lifetime compatibility with power transformers. Besides that, an aluminum enclosure was produced with an air-gap of 10 cm between the back of the structure and the antenna ground plane. This distance is necessary to avoid interference, as demonstrated in Section 3.2. Figure 9 shows a photograph of the manufactured UHF sensor, where (a) the radiating element of the bio-inspired antenna; (b) the ground plane on the other side; (c) the aluminum sensor enclosure; and (d) the complete assembly of the UHF sensor are shown.

## 4. Experimental Testing

For the sensor functionality verification, two sets of measurements were performed. The first one concerns the obtaining of the sensor electrical parameters. The second one was the PD measurement test.

### 4.1. Electrical Parameters

**Figure 10:**
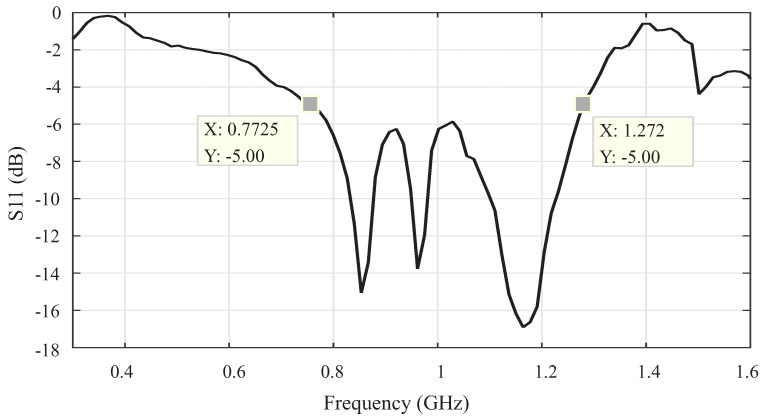
Reflection coefficient of the UHF sensor.

To obtain the main electrical parameters of the sensor, measurements were performed in an anechoic chamber using a VNA E5071C (9 MHz–8.5 GHz) from Keysight Technologies. Initially, the sensor reflection coefficient was obtained, the result is shown in Figure 10.

As can be seen in Figure 10, the UHF sensor presented a higher reflection coefficient than the bio-inspired antenna. This result was expected because the UHF sensor aluminum enclosure creates a coupling effect that interferes with the microstrip patch. However, despite the degeneration of the reflection coefficient, the sensor bandwidth can be based on the −5 dB limit of the measured $S_{11}$, which represents a transmission of approximately 70% of the power signal. After these considerations, the sensor operation is observed in the frequency range of 772 to 1272 MHz.

**Figure 11:**
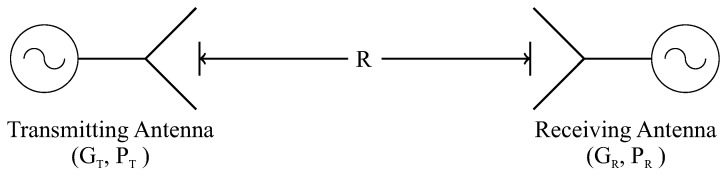
Schematic of the experimental arrangement applied on the gain measurement test.

The sensor gain was also measured. For that, an Aaronia Hyperlog 30100X was used as the reference antenna, which operates in the frequency range of 400 MHz to 10 GHz with a mean gain of 4.5 dBi. The reference antenna and the UHF sensor were positioned in front of each other at a distance (R) of 1.75 m, defined according to the far-field (Fraunhofer) region of the UHF sensor, as illustrated in Figure 11.

**Figure 12:**
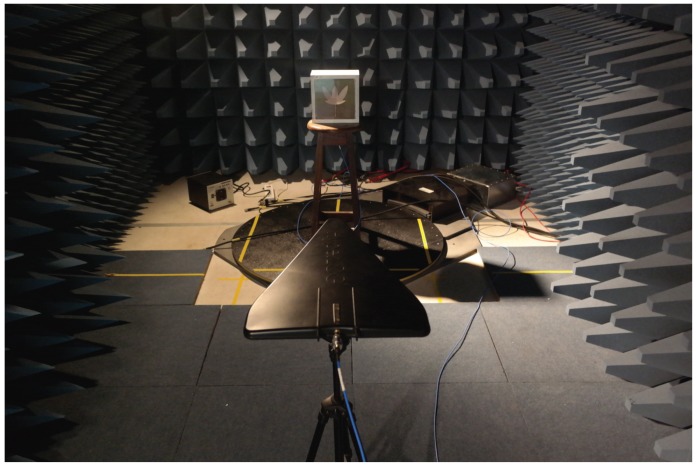
Gain measurement setup.

From the measured values of transmitted and received powers, the gain of the UHF sensor was calculated according to the Friis Transmission Equation [44]:

(3) $${\left[G_{R}\right]}_{dB}={\left[P_{R}\right]}_{dB}-{\left[P_{T}\right]}_{dB}-{\left[G_{T}\right]}_{dB}-[1-|\Gamma_{R}{|}^{2}{]}_{dB}-[1-|\Gamma_{T}{{|}^{2}]}_{dB}+20log_{10}\left(\frac{4\pi R}{\lambda }\right)$$

where $G_{R}$ and $G_{T}$ are the gains of the reference and the test antenna, respectively, and $P_{R}$ and $P_{T}$ are the measured powers of the reference and the test antenna, respectively. The terms $\Gamma_{R}$ and $\Gamma_{T}$ are the return losses of the reference and the test antenna, respectively. The term ${\left(\lambda /4\pi R\right)}^{2}$ is denominated the free-space loss factor, and it takes into account the losses as a result of the spherical spreading of the energy by the antenna. A photograph of the gain measurement setup is shown in Figure 12.

**Figure 13:**
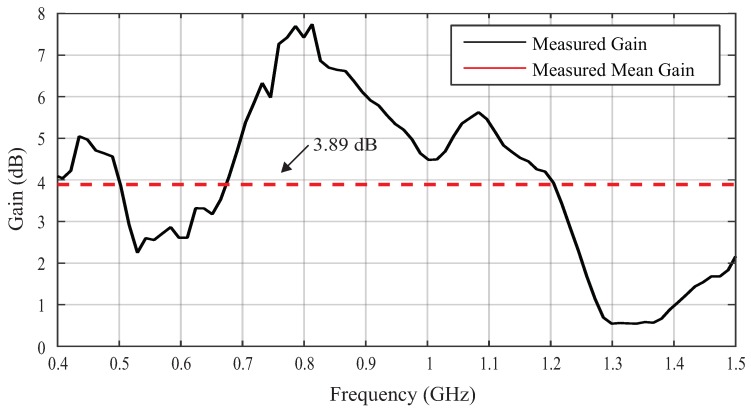
Measured UHF sensor gain.

The first result obtained is the gain of the UHF sensor as a function of the frequency, as shown in Figure 13. It can be observed that the UHF sensor has a positive gain in most parts of the frequency band and a mean gain of 3.89 dB. Previous measurements with UHF antennas demonstrate that a mean gain higher than 2 dB is already sufficient for the detection of PD signals [45].

### 4.2. PD Measurement

**Figure 14:**
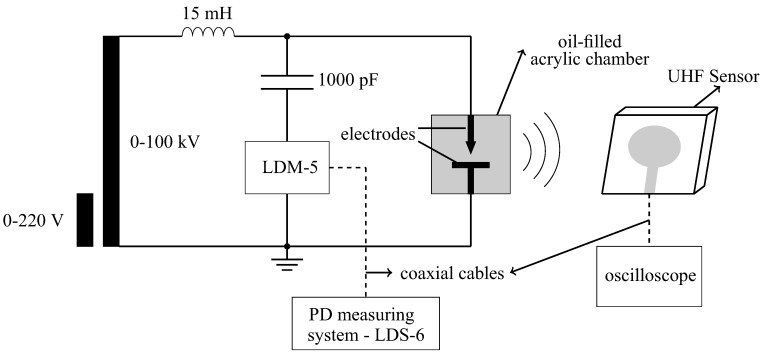
Measurement setup used for PD detection.

The third test performed was the PD measurement using the experimental setup shown in Figure 14. The setup is composed of the UHF sensor; a coupling capacitor of 1000 pF; an inductor of 15 mH; a measuring impedance LDM-5 and a digital PD measuring system LDS-6, both developed by Double Lemke; and an oil-filled acrylic chamber with electrodes for PD generation. The measurements were carried out using a Keysight oscilloscope DSO90604A with bandwidth of 6 GHz, sampling rate of 20 GSa/s, rise time of 70 ps and four analog channels. To minimize attenuation of the UHF signal in the coaxial cable, high efficiency coaxial cables, model SPUMA 400-FR-01, with attenuation of 0.13 dB/m, were used. The procedure adopted for the PD measurement levels was defined according to the IEC 60270 standard [1].

**Figure 15:**
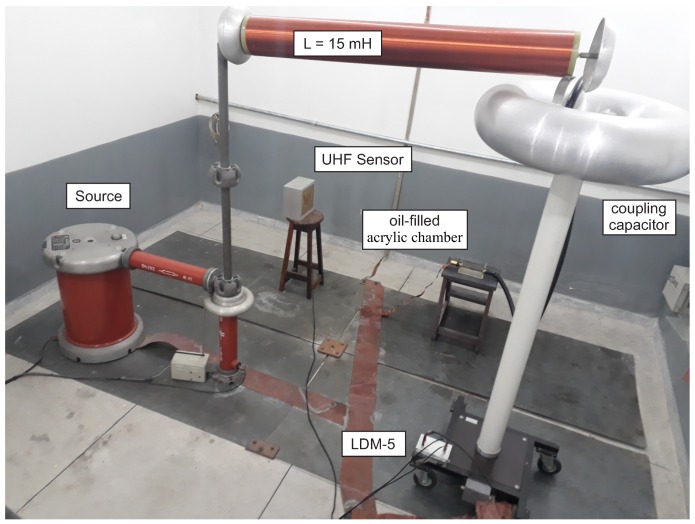
Photograph of the PD measurement setup.

**Figure 16:**
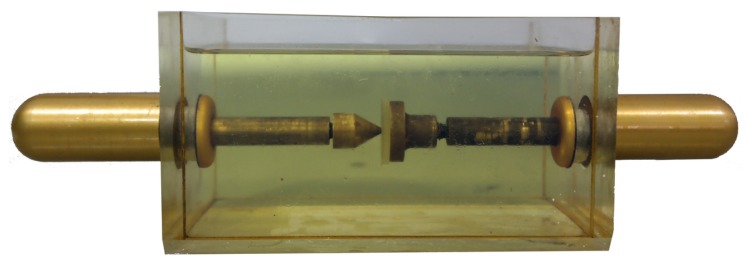
Photograph of the electrodes used for the PD production.

In order to produce the PD signals, a set of needle-plane electrodes and a polyamide disk (PA) were used. The PA disk was inserted between the electrodes and immersed in oil, in order to produce PD of low intensities. The Figure 15 and Figure 16 show photographs of the measurement setup and the electrodes used for the PD production, respectively.

**Figure 17:**
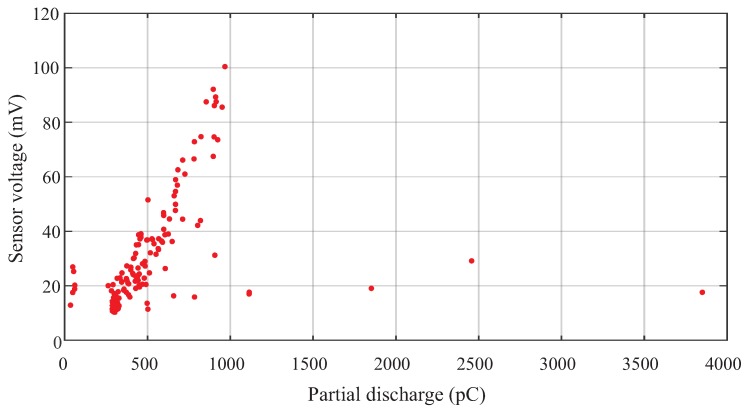
Correlation between the PD signal detected by the UHF sensor and the IEC 60270 standard method for the distance of 1 m between the UHF sensor and the PD source.

With the measurement setup used, it was possible to obtain PD levels within a wide range, depending on the applied voltage level. Figure 17 shows, in the form of a correlation plot, the measured apparent charge of the PD pulses and the voltage obtained on the UHF sensor. After several tests, it was verified that the UHF sensor was able to identify PD signals of 35 pC at a distance of 1 m from the PD source. Higher levels of up to thousands of picocoulombs were also obtained.

**Figure 18:**
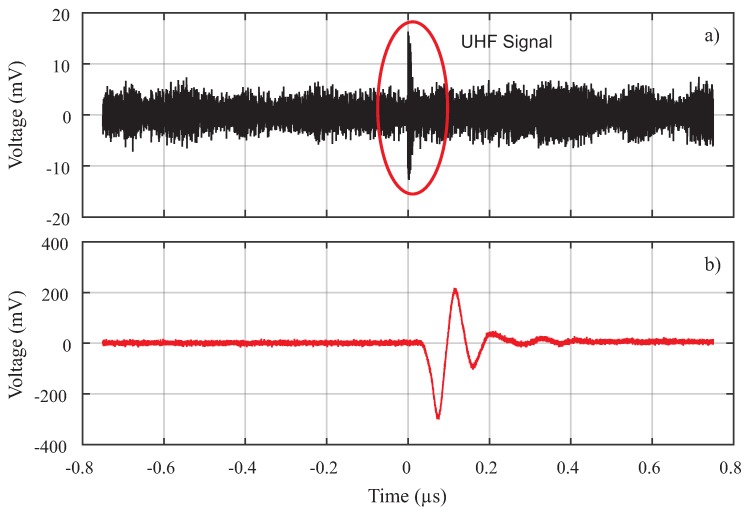
Comparison between the PD signal detected by (**a**) the UHF sensor and (**b**) the IEC 60270 standard method.

To illustrate a signal obtained by the UHF sensor, in Figure 18, a signal measured on the sensor and the same signal measured directly on the measuring impedance device are shown. As can be seen, the UHF signal is clearly distinguishable from the white noise present in the measurement and it has a typical PD shape, with a pulse shorter than the electrical signal obtained in the measuring impedance. Furthermore, it is observed that the UHF signal presented a time shift due to the signal propagation velocity to the UHF sensor, which was higher than the one noticed in the IEC 60270 standard setup.

## 5. Discussion

From the obtained results, some considerations can be made about the research developed in this paper.

First, the bio-inspired antenna was originally designed to obtain the reflection coefficient below −10 dB. However, as a result of the electromagnetic shielding effect caused by the aluminum enclosure, there was a reduction in the coefficient to approximately −5 dB. As discussed previously, this reduction in the signal transmission capacity is a known effect, since the sensor aluminum enclosure creates a coupling effect that interferes with the microstrip patch. However, a limit of −5 dB in the reflection coefficient for the definition of the antenna bandwidth is sufficient for the most applications, since it allows transmission of approximately 70% of the power signal. In other studies, such as References [46] and [47], antennas with an even higher coefficient of reflection, in the order of −3.5 dB, were satisfactory for PD detection. Therefore, the sensor aluminum enclosure presented more advantages, especially the electromagnetic shielding, than disadvantages.

Second, the UHF sensor has demonstrated its effectiveness for PD detection in power transformers. The detected sensitivity for PD levels higher than 35 pC is satisfactory, since commissioning tests allow PD levels of up to 200 pC during a 1 minute overpotential test at 1.6 times the normal operating voltage, according to the procedures established in the IEC 60076 standard [48]. In addition, according to Reference [49], depending on the voltage and size of the transformer, levels up to 500 pC are acceptable for normal operating voltage. Therefore, the UHF sensor developed in this research has shown satisfactory sensitivity for the expected application.

Finally, it can be affirmed that the methodology for the development of a UHF sensor for power transformers with a bio-inspired microstrip antenna is very promising, since the developed UHF sensor was demonstratively effective. One of the advantages of this type of sensor is its easy manufacturing and low cost, with the possibility of using other bio-inspired shapes to optimize one or more parameters of the sensor. Moreover, these advantages are associated with protection against corrosion guaranteed by the epoxy coating and the mechanical strength produced by the aluminum enclosure of the sensor. In this way, the use of the sensor against dielectric windows is guaranteed for the lifetime of the transformer.

## 6. Conclusions

In this work, a step-by-step procedure was presented for the design of a UHF sensor for use in dielectric windows of power transformers. Three main contributions were presented. Firstly, we have the development of a UHF sensor with four main parts: (1) one microstrip antenna with bio-inspired technology, to capture the UHF signal radiated from PD sources; (2) an epoxy coating, which provides protection against corrosion, mechanical support, and high temperature tolerance; and (3) an electromagnetic shield, in order to guarantee immunity from external noise. The developed sensor has an average gain of 3.89 dB and it demonstrated its effectiveness for the detection of PD signals with apparent charge values higher than 35 pC. In addition, it is designed to have a lifetime compatible with that of power transformers.

The second main contribution was the detailed analysis of the effect of electromagnetic shielding on the parameters of microstrip antennas. After analysis, it was found that a distance of at least 10 cm was required between the evaluated antenna ground plane and any metal structure to minimize bandwidth losses.

The third main contribution was the methodology, which was demonstrated as being very effective. With this, other UHF sensors for use in electrical equipment can be developed using the same methodology, with the possibility of using other bio-inspired shapes to optimize one or more of the sensor’s parameters.
